# Outpatient pharmacologic weaning for neonatal abstinence syndrome: a systematic review

**DOI:** 10.1017/S1463423618000270

**Published:** 2018-05-09

**Authors:** Jodie Murphy-Oikonen, Karen McQueen

**Affiliations:** 1 Assistant Professor, Lakehead University School of Social Work, Thunder Bay, ON, Canada P7B 5E3; 2 Associate Professor, Lakehead University School of Nursing, Thunder Bay, ON, Canada P7B SE3

**Keywords:** home weaning; neonatal abstinence syndrome; outpatient treatment; outpatient weaning; pharmacologic treatment

## Abstract

**Aim:**

The purpose of this systematic review was to assess the literature regarding the effectiveness and safety of outpatient pharmacologic weaning for infants with neonatal abstinence syndrome (NAS).

**Background:**

NAS is a multi-system disorder observed in infants experiencing withdrawal from opioid exposure *in utero*. Infants requiring pharmacologic treatment to manage withdrawal, traditionally receive treatment as a hospital inpatient resulting in lengthy hospitalization periods. However, there is evidence to suggest that some healthcare institutions are continuing outpatient pharmacologic weaning for select infants in a home environment. As there is no standard of care to guide outpatient weaning, assessment of the safety and effectiveness of this approach is warranted.

**Method:**

A systematic review of outpatient weaning for infants with NAS was conducted using the electronic databases PubMed, Nursing and Allied Health, CINAHL, Evidence-Based Medicine, Web of Science, Medline, and PsychINFO. Studies were eligible for inclusion in the review if they fulfilled the following criteria: (1) reported original data on outcomes related to the effectiveness or safety of outpatient weaning for infants with NAS, (2) infants were discharged from hospital primarily receiving opioid pharmacologic treatment for NAS, (3) the method included quantitative designs that included an inpatient comparison group, and (4) articles were published in English in a peer-reviewed journal.

**Findings:**

The search identified 154 studies, of which 18 provided information related to NAS and outpatient weaning. After reviewing the remaining full-text studies, six studies met all inclusion and exclusion criteria. All studies identified that outpatient weaning for select infants was associated with shorter hospitalization compared with infants weaned in-hospital only and may be potentially effective in reducing associated healthcare costs. However, duration of pharmacologic treatment was longer in the outpatient weaning groups in the majority of the studies. Furthermore, adverse events were rare and compliance to follow-up treatment was high among those who received outpatient weaning.

## Introduction

Neonatal abstinence syndrome (NAS) is a multi-system disorder observed in infants experiencing withdrawal from opioid exposure *in utero* (Stover and Davis, 2015; McQueen and Murphy-Oikonen, 2016). Care of infants typically begins with non-pharmacologic comfort measures to decrease the severity of symptoms and mitigate negative neonatal outcomes (Maguire, 2014; Edwards and Brown, 2016). However for infants who do not respond, pharmacotherapy may be warranted to manage the symptoms of withdrawal (McQueen and Murphy-Oikonen, 2016). Traditionally, pharmacologic treatment for infants with NAS is completed in-hospital until infants are stable and fully weaned from medication. However, there is some evidence to suggest that some hospitals may continue an outpatient weaning regimen (Napolitano *et al.*, 2013; Chau *et al.*, 2016), as many infants with NAS are otherwise healthy (Burns *et al*., 2007; O’Grady *et al*., 2009). This type of combined inpatient/outpatient treatment may assist to alleviate the strain of NAS on the healthcare system and decrease separation of mothers and infants. However, there is currently no consensus or standard of care to guide outpatient weaning (Chau *et al*., 2016; Patrick *et al*., 2016). Understanding the evidence regarding outpatient weaning for NAS is an important consideration for organizations seeking to adopt this method. To date, there are no systematic reviews available on outpatient weaning for NAS. Thus, the purpose of this systematic review was to assess the existing literature regarding the effectiveness and safety of outpatient pharmacologic weaning on NAS outcomes for infants with NAS. The specific review question was: Among infants with NAS who require pharmacologic treatment, what is the effect of a combined inpatient/outpatient weaning (eg, home weaning) versus in-hospital weaning on NAS outcomes (eg, length of stay, duration of treatment, cost, breastfeeding) and infant safety.

NAS presents as central nervous system hyperirritability, autonomic nervous system dysfunction, and gastrointestinal disturbances (Kocherlakota, 2014; Stover and Davis, 2015). Defining characteristics of the syndrome include a high pitched cry, irritability, fever, tremors, excessive sucking, weight loss, loose stools, poor feeding, sleep disturbances, excoriation of the skin, respiratory distress, and seizures (Finnegan *et al*., 1975). Clinical manifestations of the syndrome are variable and are based on the type of opioid, timing of exposure, and maternal and placental metabolism (Hudak and Tan, 2012). Initial signs of NAS are often observed within the first 24–72 h (Hudak and Tan, 2012; Kaltenbach and Jones, 2016); with most infants demonstrating signs of withdrawal within 12 h of birth (Kaltenbach and Jones, 2016).

The incidence of NAS has risen dramatically in the United States. Between the years 2000 and 2009 the incidence of NAS increased from 1.20 (95% CI: 1.04–1.37) to 3.39 (95% CI: 3.12–3.67) infants per 1000 hospital births annually (Patrick *et al*., 2016). In 2013, a total of 4% of neonatal intensive care unit (NICU) admissions across the United States were attributed to a diagnosis of NAS (Tolia *et al*., 2015). Similar increases in the incidence of NAS have been reported in Canada (Davies *et al*., 2016) and across Western Australia (O’Donnell *et al*., 2009), indicating the impact from a global perspective. The higher rates of NAS correspond to the increased incidence of maternal opioid use during pregnancy (Epstein *et al*., 2013; Krans *et al*., 2015), inclusive of illicit opioid use (Cicero *et al*., 2015), prescribed opioids for pain (Ailes *et al*., 2015; Warren *et al*., 2015), and the rise in opioid replacement therapies for pregnant women with addictions (Jansson *et al*., 2009; O’Grady *et al*., 2009).

Numerous negative outcomes have been associated with NAS including admission to a special care nursery (Tolia *et al*., 2015; Uebel *et al*., 2015), a lengthy hospitalization period (Wachman *et al*., 2011; Lee *et al*., 2015), and separation of mother and infant at a critical time for bonding (Abrahams *et al*., 2010; Wiles *et al*., 2014). Lengthy periods of hospitalization are often required due to the need for pharmacologic management of withdrawal symptoms (Jansson *et al*., 2009; Hudak and Tan, 2012). Furthermore, decreased rates of breastfeeding (Wachman *et al*., 2010; Tsai and Doan, 2016) and involvement in the child protection system (O’Donnell *et al*., 2009) are additional negative outcomes associated with NAS.

Although not all substance-exposed infants require pharmacologic treatment, a substantial number (60–80%) do require treatment to manage withdrawal (Kocherlakota, 2014). Pharmacologic treatment is diverse and is often contingent on physician practices or specific organizational protocols (Hall *et al*., 2015) as no universal pharmacologic treatment has been established. Methadone or oral morphine are recommended as a first-line pharmacologic treatment for opioid withdrawal, although clonidine and buprenorphine have also been administered to manage the symptoms (McQueen and Murphy-Oikonen, 2016).

Recent studies identify that the practice of continuing pharmacologic weaning for NAS out of the hospital setting has been implemented in some healthcare institutions (Saunders *et al*., 2014; Kaltenbach and Jones, 2016; Kraft *et al*., 2016). In particular, in a study evaluating quality improvement for NAS, Patrick *et al.* (2016) found that 34% of infants from 199 centers were discharged from the hospital on medication to be weaned in a home environment. However, there is no consensus or standard of care to guide outpatient weaning (Chau *et al*., 2016; Patrick *et al*., 2016). Thus, this systematic review was conducted to assess the effectiveness and safety of outpatient weaning for infants with NAS. Effectiveness was determined by synthesizing the results of studies reporting between group comparisons (inpatient versus outpatient weaning) on NAS outcomes. In addition, safety was assessed by making between group comparisons on variables such as adverse events, child welfare involvement, compliance with follow-up, and hospital readmission rates.

## Methods

### Search strategy

The electronic databases PubMed, Nursing and Allied Health, CINAHL, Evidence-Based Medicine, Web of Science, Medline, and PsychINFO were searched from 1996 to October, 2017. Subject terms used in the search strategy included ‘neonatal abstinence syndrome’ (Mesh) and one of the following additional terms, outpatient treatment, home treatment, or outpatient weaning. To ensure relevant articles had not been missed, the reference lists of included studies were reviewed for additional articles relevant to the initial search.

### Study selection

The review followed the Preferred Reporting Items for Systematic Reviews and Meta-analysis (PRISMA) statement (Moher *et al*., 2009). Studies were eligible for inclusion in the review if they fulfilled the following criteria: (1) reported original data on outcomes related to the effectiveness or safety of outpatient weaning for infants with NAS, (2) the infants were discharged from hospital primarily receiving opioid pharmacologic treatment for NAS, (3) the study method included any type of quantitative design that included an inpatient comparison group (infants receiving pharmacologic treatment for NAS in-hospital only), and (4) the articles were published in English in a peer-reviewed journal. For the purpose of this review, NAS was defined as a postnatal withdrawal syndrome in infants that were exposed to opioids *in utero* (McQueen and Murphy-Oikonen, 2016). Thus, NAS in infants exclusively from substances other than opioids (eg, selective serotonin reuptake inhibitors) were excluded from this review. Additional exclusion criteria included: (1) infants being treated for NAS with paregoric, tincture of opium or diazepam as they are not currently recommended for treatment of NAS and (2) infants who were readmitted to hospital with NAS after discharge. Outpatient weaning refers to infants who were initiated on pharmacologic treatment in hospital and received continued pharmacologic treatment for weaning as an outpatient.

**Fig. 1:**
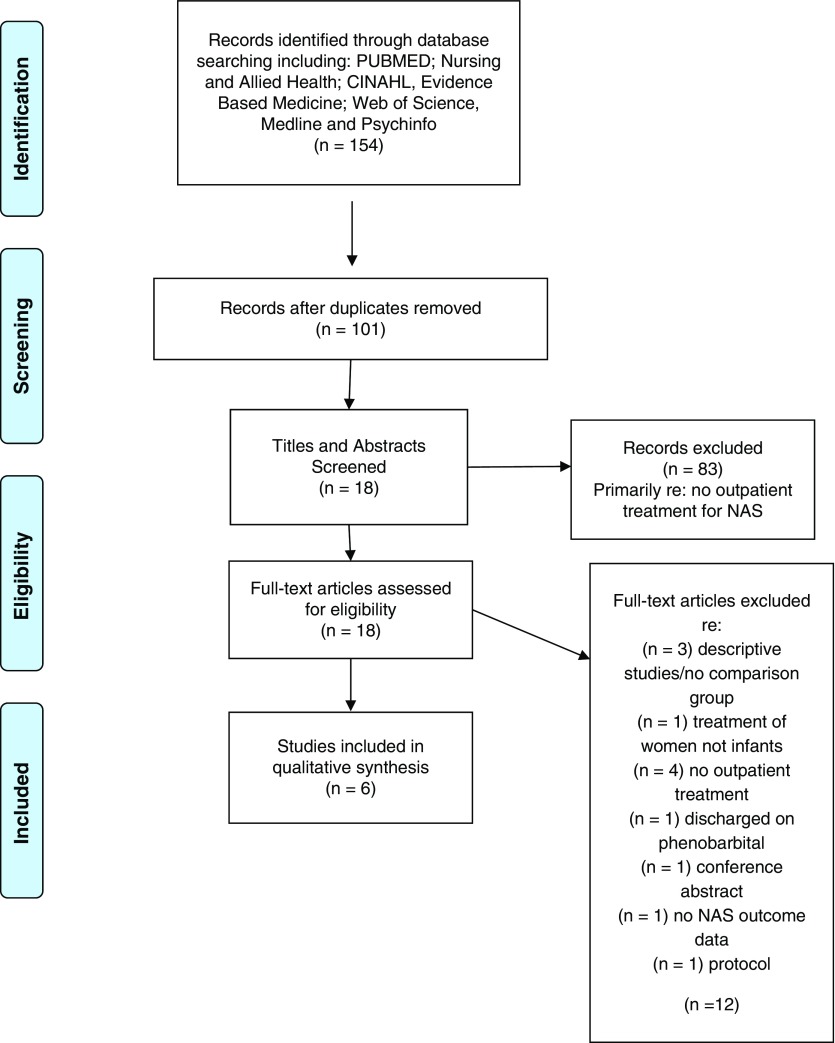
Preferred Reporting Items for Systematic Reviews and Meta-analysis (PRISMA) flow diagram: outpatient weaning

The first author entered all studies from the search into the Zotero Reference Manager. Duplicates were removed and remaining studies were screened for inclusion based on the title, abstract and full text (see Figure 1). Articles that did not meet the inclusion and exclusion criteria were eliminated for further review. The second author screened the excluded articles to ensure accurate exclusion.

### Data extraction

Data from full-text studies was extracted by the principal author and entered into a data extraction template developed for the systematic review in order to capture all relevant details. The template included the authors’ names, date of publication, purpose, study design, number of participants, pharmacologic treatment type, neonatal outcomes, and safety data. The second author also independently extracted data onto a template. The extracted data were then compared and differences were discussed, referring back to the article until an agreement was obtained. A research assistant reviewed the final data extraction template for accuracy or omissions. The data were synthesized narratively as meta-analysis was not possible due to the heterogeneity of the included study samples and outcomes evaluated.

### Assessment of methodological quality

Articles selected for inclusion were assessed for methodological quality by two independent reviewers (K.M. and a graduate student) using the Joanna Briggs Institute (JBI) standardized critical appraisal checklist for cohort studies (Joanna Briggs Institute, 2014). Studies were rated as having a low, moderate, or high risk of bias based on participant selection, measurement of exposure (NAS) and outcomes, confounding factors, and follow-up. The independent assessments were compared and any disagreements were resolved through discussion with the primary author (J.M.O.). No studies were eliminated based on the critical appraisal.

## Results

The search identified 154 studies, of which 18 provided information related to NAS and outpatient weaning. Further assessment of the inclusion and exclusion criteria eliminated 12 of the studies due to: descriptive studies/no comparison group (*n*=3); treatment of women not infants (*n*=1); no outpatient treatment (*n*=4); discharged on phenobarbital (*n*=1); no NAS outcome data (*n*=1); conference abstract (*n*=1); and published protocol (*n*=1). A total of six articles met all criteria and are included in the review. See Figure 1 for the flow diagram.

### Study characteristics

The characteristics of the six included studies are provided in Table 1. All of the studies were retrospective chart reviews published between 2012 and 2015 and were conducted in the United States (*n*=3), Canada (*n*=1), and Australia (*n*=2). For three of the studies, the primary purpose was to evaluate outpatient weaning for infants with NAS. Whereas the other three studies reported on a subset of infants who received outpatient weaning, although it was not the primary purpose or outcome of the study. Sample sizes ranged from 80 to 981, with a median of 130 participants. Recruitment settings were hospitals that provided care for infants with NAS.

**Table 1:** Characteristics of included studies

Author	Purpose of study	Population/setting	Outpatient weaning	Research design	Quality appraisal/risk of bias assessment
Abdel-Latif *et al*. (2013)	To ascertain the characteristics and short-term outcomes of infants born to illicit drug-using mothers	871 infants born to drug-dependent mothers. 202 infants required pharmacologic treatment for NAS at public hospitals in the state of New South Wales, Australia	Did not provide specific information regarding criteria or treatment for outpatient weaning	Retrospective audit of clinical records	Moderate
Backes *et al*. (2012)	To compare the safety and efficacy of a traditional inpatient only treatment versus a combined inpatient and outpatient weaning strategy for infants with NAS	244 infants born to women on methadone. 121 of these infants required pharmacologic treatment for NAS at Ohio State University Medical Centre, USA	Infants followed a stringent screening protocol to assess inclusion in the home weaning group including medical and social clearance and a physician/caregiver agreement. NAS follow-up care was provided in an outpatient clinic staffed by physicians, nurses, and an interprofessional team of social workers, lactation specialists, physical/occupational therapists and a pharmacist	Retrospective review	Moderate
Hall *et al*. (2015)	To evaluate the generalizability of a stringent protocol-driven weaning program for infants with NAS. Additional outcomes included one hospital that conducted outpatient weaning and compared to in-hospital treatment of NAS	981 infants pharmacologically treated for NAS from 20 hospitals, across six regions in Ohio, USA. A subset of 117 infants were discharged on home weaning	Before discharge, approval was required from social services and a family support system needed to be in place to reliably administer medications. Infants were followed by a pediatrician and regular nursing home visits	Retrospective cohort study	Low
Kelly *et al*. (2015)	To evaluate the safety and effectiveness of discharging stabilized neonates to complete oral morphine weaning at home	80 neonates required pharmacologic treatment for NAS from two hospitals in London, ON, Canada	Infants were considered for home weaning if social stability was demonstrated and caregivers were judged to be competent in administering morphine doses. Caregivers were trained in administration, provided with a case worker, and taught non-pharmacologic strategies for NAS	Retrospective cohort study	Moderate
Lee *et al*. (2015)	To examine NAS records from a large Medicaid database regarding average length of stay and variables affecting average length of stay. Secondary outcome explored length of stay and contributing variables including treatment regimen	139 infants required pharmacologic treatment for NAS from 22 hospitals in Pennsylvania, USA	Did not provide specific information regarding criteria or treatment for outpatient weaning	Retrospective data analysis	High
Smirk *et al*. (2014)	To evaluate the safety and efficacy of a home-based detoxification program compared with standard inpatient care for infants affected with NAS	118 infants with NAS during the study period (2004–2010) at Royal Women’s Hospital (tertiary perinatal hospital), Australia	Home weaning was available to eligible infants once one dose reduction had been tolerated. Assessment completed by social worker before discharge. Follow-up for infants receiving outpatient weaning included nursing visits and phone calls and outpatient visits by a neonatologist	Retrospective review	Moderate

NAS=neonatal abstinence syndrome.

The studies evaluated within this review outlined various practices in the care of infants with NAS. Most studies identified a detailed discharge plan for infants beginning a home weaning program. While all of the studies that indicated the use of a discharge plan required established medical follow-up from a neonatologist or pediatrician, the remaining elements were diverse. The hospital practices included medical stabilization (Backes *et al*., 2012; Smirk *et al*., 2014; Hall *et al*., 2015); social stability established by social work (Backes *et al*., 2012; Smirk *et al*., 2014; Hall *et al*., 2015; Kelly *et al*., 2015); a physician/caregiver agreement (Backes *et al*., 2012) confirmed family and social support (Hall *et al*., 2015; Kelly *et al*., 2015); and nursing home visits or calls post-discharge (Smirk *et al*., 2014; Hall *et al*., 2015).

The primary medications used for pharmacologic treatment of NAS were morphine (*n*=4), methadone (*n*=1), or either morphine or methadone (*n*=1) (see Table 2). Phenobarbitone was used as monotherapy for a very small number of infants (eg, <5%), and as a second (eg, adjunctive) medication during home weaning in two studies (Abdel-Latif *et al*., 2013; Smirk *et al*., 2014). The Finnegan or modified version of the Finnegan Scoring tool was used to assess symptoms of NAS and guide treatment in all of the studies. However, it was unclear if they were the same tools as the number of items and/or modifications were not specified. This reflects the diversity of the studies included in the systematic review in terms of treatment (medication, weaning protocols) and outcome measures and participants (eg, mothers in treatment, polysubstance use, term/premature infants). Outcomes measured for the systematic review included: (1) length of hospital stay, (2) duration of treatment, (3) cumulative dose of pharmacologic treatment, (4) healthcare costs, (5) breastfeeding, (6) adverse events, and (7) follow up/child welfare involvement.

**Table 2:** Synopsis of weaning and outcomes

Author/date	Home weaning treatment	In-hospital treatment	Effect on NAS outcomes	Infant safety outcomes
Abdel-Latif *et al*. (2013)	Infants were discharged home on medication (*n*=143), primarily morphine	Infants were weaned in hospital (*n*=59), primarily morphine	Decreased length of stay among infants weaned at home (median 12 days) versus in-hospital (median 26 days) *P*<0.001	Study did not assess safety outcomes for home weaning and/or inpatients
Backes *et al*. (2012)	Infants were discharged for outpatient weaning (*n*=46) with methadone	Infants (*n*=75) received traditional inpatient only pharmacologic treatment with methadone	Decreased length of hospital stay for outpatient weaning (13 days versus 25 days) (*P*<0.01) Longer duration of treatment for outpatient group (37 days versus 21 days) (*P*<0.001) Similar cumulative dose of morphine between groups (3.6 versus 3.1) (*P*=0.42) Increased rates of breastfeeding at hospital discharge for outpatient group (24% versus 8%) (*P*<0.01). Average hospital cost for each infant was $13 817 for outpatient group compared with in-hospital ($27 546)	No short-term adverse events. No infant deaths
Hall *et al*. (2015)	Infants were discharged for outpatient weaning (*n*=117) with prescribed opioid therapy after stabilization and several consistent weans	Infants were treated in-hospital (*n*=864) with morphine or methadone using a stringent weaning protocol	Outpatient weaning had shorter length of stay (14.7 days) compared with in-hospital (existing sites 23.2 days and adopting sites 21.2 days). Duration of treatment was longer for home weaning (26.3 days) compared with in hospital (existing sites 17.3 days and adopting sites 15.7 days)	Infants followed for outpatient weaning were reported to have nearly 100% compliance with follow-up and >90% compliance with medication administration. No adverse outcomes or child protection outcomes were reported
Kelly *et al*. (2015)	Infants were discharged for outpatient weaning (*n*=52) on oral morphine	Infants were treated in-hospital (*n*=28) with morphine according to a protocol, which included oral morphine or continuous infusion if IV access was present	Outpatient weaning had significantly shorter length of stay (16 days versus 22 days, *P*=0.004). Neonates weaned at home were on oral morphine longer (32 versus 19, *P*<0.001) and were more likely to have adjuvant treatment in NICU (31% versus 4%, *P*<0.0030. Decreased healthcare costs. No significant differences were reported in breastfeeding initiation (41% outpatient versus 33% in-hospital between groups	Fewer infants had to return to hospital for withdrawal treatment among home weaning group (1/52 versus 4/28, *P*=0.044). No significant difference was found in CAS apprehension between groups (home weaning 31%, hospital weaning 39%, *P*=NS). One case of SIDS attributed to bed sharing among home weaning group and unsafe sleeping environment. No other safety concerns identified
Lee *et al*. (2015)	Infants were discharged for outpatient weaning (*n*=37) with methadone as part of standard treatment for NAS for period A from 2007 to April 2013.	Standard treatment for NAS changed to inpatient only treatment. Infants were treated in hospital (*n*=6) with morphine as standard treatment changed to in-hospital in May–Dec 2013 (period B)	Length of stay was lower for infants receiving outpatient weaning. The average length of stay for outpatient weaning varied yearly between 7.3 and 15.7 days from 2007 to April 2013) compared with those treated solely as an inpatient with morphine May–Dec 2013 (37.5 days). Decreased healthcare costs among home weaning group	One readmission for an infant discharged on home weaning due to worsening NAS symptoms due to inability to obtain methadone doses
Smirk *et al*. (2014)	Infants were discharged for outpatient weaning (*n*=38) primarily with morphine (*n*=35) or phenobarbitone (*n*=3)	Infants (*n*=80) treated in hospital only with morphine (*n*=78), phenobarbitone (*n*=2), or both (*n*=9)	Decreased length of hospital stay among home weaning group 18.9 versus 39.6 days (*P*<0.001) Shorter duration of treatment 36.1 versus 42.0 days (*P*=0.016) Total dose of morphine (mg/kg/birth weight) was lower for those weaned at home (10.2) versus in-hospital (11.4) (*P*=0.28). Increased breastfeeding at discharge 45% versus 22% of newborns (*P*=0.022) Decreased need for treatment with phenobarbitone 10.5% versus 13.8% (*P*=0.62)	Decreased need for child welfare involvement 24% versus 68% (*P*<0.001) among outpatient weaning group Similar frequency in adverse events Two medication errors were reported among home weaning group. One infant in inpatient group was readmitted for withdrawal symptoms, but no treatment was required

NAS=neonatal abstinence syndrome; NICU=neonatal intensive care unit; CAS=children’s aid society; SIDS=sudden infant death syndrome.

### Methodological quality

Overall, the majority (*n*=4) of the included studies were identified to have a moderate risk of bias (Backes *et al*., 2012; Abdel-Latif *et al*., 2013; Smirk *et al*., 2014; Kelly *et al*., 2015) (see Table 1). One study was identified at low risk of bias (Hall *et al*., 2015) and one with high risk of bias (Lee *et al*., 2015). Selection bias was present among all studies as the groups were not similar at the outset. Most of the studies identified that infants receiving home weaning had to meet certain discharge criteria for eligibility, which typically included family stability. Likewise, a lack of reporting and/or controlling for confounding variables was present in the majority of studies. Many of the studies reported on baseline characteristics of infants and mothers such as gestation, birth weight, smoking that may affect NAS symptoms; however, other potential confounding factors such as maternal drug and non-pharmacologic treatment for NAS were not controlled for in the analysis. While all studies identified using the Finnegan Scoring Tool or Modified Finnegan to assess NAS and guide treatment, no studies reported on psychometric data regarding the reliability or validity of the tool. Finally, for many of the outcomes it was unclear whether there was a loss to follow-up and how many infants were included in the final analyses.

### Home weaning and NAS outcomes

Of the six studies included, all reported on one or more NAS outcomes including length of hospital stay, duration of treatment, healthcare costs and breastfeeding (see Table 2). All studies found that there was a significantly reduced length of hospital stay associated with home weaning. Among infants who received outpatient weaning, the shortest length of stay was 7.3 days (Lee *et al*., 2015) and longest was 18.9 days (Smirk *et al*., 2014). For infants who received only in-hospital weaning, the length of stay was much longer, ranging from 15.7 days (Hall *et al*., 2015) to 39.6 days (Smirk *et al*., 2014). With the decreased length of stay, considerable cost savings were reported among the three studies that evaluated healthcare expenditures (Backes *et al*., 2012; Kelly *et al*., 2015; Lee *et al*., 2015).

While length of stay was reduced, the total duration of opioid treatment for infants who received the outpatient weaning was often longer. In three out of four studies, researchers found that infants who received home weaning received opioid treatment for a longer time period compared with in-hospital treatment (Backes *et al*., 2012; Hall *et al*., 2015; Kelly *et al*., 2015). Only, Smirk *et al.* (2014) found that the total length of treatment was lower for home weaning infants (36.1 days) compared with in-hospital (42 days, *P*=0.016). Despite the variations in the duration of treatment among studies, no significant differences were found between groups related to cumulative dosage of morphine (Backes *et al*., 2012; Smirk *et al*., 2014). Smirk *et al*. (2014) found a lower cumulative dose of morphine (mg/kg/birth weight) for those weaned at home (10.2) compared to in-hospital (11.4), although not statistically significant (*P*=0.28). Whereas Backes *et al*. (2012), who reported longer treatment duration for outpatient weaning (37 versus 21 days, *P*=0.001), found no significant differences between groups regarding the cumulative morphine doses (mg/kg) (*P*=0.42).

Three studies reported on rates of breastfeeding. In two of the studies, researchers identified significant differences in breastfeeding rates between groups (Backes *et al*., 2012; Smirk *et al*., 2014). Smirk *et al*. (2014) found that infants in the outpatient weaning group were almost three times more likely to have been breastfeed upon discharge (*n*=15, 45%) compared with infants in the hospital-based group (*n*=18, 22%) (OR 2.9, 95% CI: 1.2–6.8). Similarly, Backes *et al.* (2012) found that discharge rates of breastfeeding were significantly higher at hospital discharge for the home weaning group (24% versus 8%, *P*<0.01). However, Kelly *et al*. (2015) found breastfeeding initiation rates were similar between groups with 17 (41%) breastfeeding in the outpatient weaning group and seven (33%) breastfeeding in the hospital only group.

### Home weaning and safety

Overall, few adverse events were associated with home weaning. No adverse events were reported in two studies (Backes *et al*., 2012; Hall *et al*., 2015), whereas Smirk *et al*. (2014) found a similar frequency of adverse events between groups. Adverse events that were reported among infants on home weaning included one readmission for NAS due to two missed methadone doses (Lee *et al*., 2015) and one case of sudden infant death syndrome attributed to unsafe sleeping (Kelly *et al*., 2015). Comparatively, infants weaned in hospital were more often readmitted for withdrawal treatment after discharge than those weaned at home (4/28 versus 1/52, *P*=0.44) (Kelly *et al*., 2015). Further, a higher proportion of infants in the hospital group required child protection involvement (OR 0.015, 95% CI: 0.06–0.36) or foster care (OR 0.13, 95% CI: 0.03–0.58) when compared with infants weaned at home.

Positive outcomes were also identified regarding outpatient follow-up and child welfare involvement for infants who received home weaning. Outpatient clinic compliance was high with nearly 100% adherence to follow-up appointments and >90% compliance with medication administration (Hall *et al*., 2015). In addition, there was a decreased need for child welfare involvement for the home weaning group compared with in-hospital (24% versus 68%, *P*<0.001) (Smirk *et al*., 2014).

## Discussion

This is the first systematic review to assess the effectiveness and safety of outpatient pharmacologic weaning for infants with NAS. Overall, all studies consistently identified that outpatient weaning for select infants, was associated with shorter hospital stays compared with infants weaned in-hospital only. These findings were consistent regardless of the pharmacologic agent used to wean (methadone, morphine) and the healthcare provider regimen for follow-up. Furthermore, three of the six studies (Backes *et al*., 2012; Kelly, 2015; Lee *et al*., 2015) reported a reduction in healthcare expenditures for infants weaned at home; however, only one study provided cost estimates of ~$11 000 in healthcare savings for each neonate (Kelly, 2015). This review also identified that adverse events were rare and compliance to follow-up treatment was high among those who received outpatient weaning.

Decreased length of hospital stay is an important development given that the length of hospital stay for infants with NAS has remained relatively unchanged in over a decade (Patrick *et al*., 2012; Tolia *et al*., 2015). This is substantial as Patrick *et al*. (2012) estimated the average costs of NAS as $53 400 per infant (95% CI: $49 000–$57 700), while Tolia reported that 4% of all NICU days across the United States are attributed to NAS. With the incidence of NAS and related healthcare expenditures on the rise (Patrick *et al*., 2012; Tolia *et al*., 2015), a reduction in the period of hospitalization for infants with NAS will invariably lead to a reduction in acute healthcare-related expenditures.

Despite the improvements to the length of hospital stay for infants receiving outpatient weaning, concerns exist regarding the longer duration of outpatient pharmacologic treatment found in three of the studies (Backes *et al*., 2012; Hall *et al*., 2015; Kelly *et al*., 2015). A longer duration of pharmacologic treatment requires careful consideration given that the long-term outcomes of prolonged opioid treatment are unclear (Hall *et al*., 2015; Kraft *et al*., 2016). However, while the duration of treatment was longer, the cumulative dose of pharmacologic treatment did not differ between the inpatient and outpatient groups in two of the three studies that measured cumulative dose (Backes *et al*., 2012; Smirk *et al*., 2014). Thus, the longer duration of treatment did not directly translate into receiving a higher dosage of the medication. The longer duration is likely reflective of a slower taper, which may be advantageous for infants receiving outpatient weaning as few infants returned to hospital for further NAS treatment (Kelly *et al*., 2015). These findings support the assertion by Hall *et al.* (2015) that the use of evidence-based protocols for the management of NAS are needed to improve neonatal outcomes. Thus, further evaluation of weaning protocols and the length of opioid treatment is needed.

Overall, this review found that serious short-term adverse events were rare (Backes *et al*., 2012; Smirk *et al*., 2014; Hall *et al*., 2015; Kelly *et al*., 2015; Lee *et al*., 2015) and there was a high rate of compliance with outpatient follow-up. Furthermore, findings revealed that infants receiving outpatient weaning more frequently remained in the care of their biological parents when compared with in-hospital only groups (Smirk *et al*., 2014). Among the majority of studies, very few child protection concerns were reported for infants weaned at home. However, one study reported a high rate of infant apprehensions in both the in-hospital and outpatient weaning group (Kelly *et al*., 2015). While all institutions had eligibility criteria for outpatient weaning, the criteria were diverse, and optimal conditions for infant safety have not been established. These are important considerations given that research has found infants with NAS are at a greater risk for involvement with the child welfare system at some point in their early development (O’Donnell *et al*., 2009).

The findings from this review identified higher rates of breastfeeding for infants who were receiving outpatient weaning for NAS (Backes *et al*., 2012; Smirk *et al*., 2014). However, the mechanism for improved breastfeeding outcomes found in these studies is unknown. Maintaining the mother–infant dyad may have positively influenced breastfeeding outcomes and/or the engagement with diverse primary care providers involved in the care of infants receiving outpatient weaning. The differences between groups may also be reflective of selection bias with mothers in the in-hospital weaning group more often using multiple substances (Smirk *et al*., 2014), which is considered a contraindication to breastfeeding (Reece-Stremtan and Marinelli, 2015). Regardless, these are noteworthy findings as breastfeeding has been associated with positive impacts on NAS outcomes including delayed symptom onset (Liu *et al*., 2015), reduced incidence, decreased severity of symptoms, and decreased pharmacotherapy compared with infants who are not breastfed (Pritham, 2013; Welle-Strand *et al*., 2013). As such, breastfeeding should be recommended as a supportive non-pharmacologic treatment for NAS among stabilized mothers (Bagley *et al*., 2014; Kaltenbach and Jones, 2016; McQueen and Murphy-Oikonen, 2016).

Despite the potential benefits associated with breastfeeding, rates remained low in this population (Backes *et al*., 2012; Smirk *et al*., 2014; Kelly *et al*., 2015). This finding is consistent with previous research reporting on low rates of breastfeeding for infants with NAS (Wachman *et al*., 2010). Many infants requiring pharmacologic treatment for NAS are treated in a special care nursery in isolation from their mothers (Tolia *et al*., 2015; Uebel *et al*., 2015; McQueen and Murphy-Oikonen, 2016), thus inhibiting exclusive breastfeeding (Flacking *et al*., 2012). Additional barriers to breastfeeding for infants with NAS may include NAS symptoms (eg, irritability, tachypnea, increased tone) and lack of information or discouragement of breastfeeding by healthcare professionals (McQueen and Murphy-Oikonen, 2016; Tsai and Doan, 2016). Infants treated in a home environment have less structural barriers to impede breastfeeding which may positively influence breastfeeding rates among infants with NAS.

Additional benefits may be associated with outpatient weaning that have not been evaluated in this systematic review. Lengthy hospitalization for infants with NAS is associated with the separation of mother and infant at a critical time for infant development and bonding (Cleary *et al*., 2011; Tolia *et al*., 2015; Uebel *et al*., 2015). Given that the postnatal period is a crucial time for maternal-infant bonding and subsequent attachment (Crouch and Manderson, 1995; Shannon *et al*., 2016), a treatment model that decreases the separation of mother and infant may positively influence the maternal-child relationship. Thus, implementing outpatient weaning for NAS has the potential to empower mothers to assume the caregiver role in a natural environment and develop a bond with their newborn, while facilitating recovery from NAS symptoms.

### Limitations

All of the included studies were retrospective in nature and relied on the accuracy of medical records. Many studies had small sample sizes of infants that received home weaning and for some studies, evaluating home weaning was not the primary purpose. Furthermore, while the included studies discuss reduced healthcare expenditures related to decreased length of hospital stay, there was a lack of clarity of how costs were measured in two of the three studies that evaluated this outcome. In addition, the critical appraisal identified that the majority of included studies were at moderate of bias. Thus, the generalizability of the findings is limited to a select group of mothers and infants who may be appropriate for home weaning. As a result of the identified limitations, results of the systematic review need to be interpreted cautiously.

### Implications for practice

The benefits of outpatient weaning for length of hospital stay and related healthcare expenditures have influenced some healthcare institutions and provider groups to explore outpatient weaning as a treatment option for select infants with NAS. The presence of few adverse events and safety concerns likely reflects the well-developed protocols used to guide primary healthcare providers’ decision making in many of the included studies. Given the social risk factors associated with substance use (Meyer *et al*., 2010; Traube, 2012), hospitals implementing outpatient weaning need to consider social stability and follow-up services available to infants with NAS. Social stability may require a psychosocial assessment from an in-hospital social worker before consideration for discharge. Follow-up services that are inclusive of pharmacologic management and/or monitoring, nursing, social, or familial supports may also be beneficial to both mother and infant. Given the diversity of approaches to establish safety criteria before discharge of infants to continue weaning in a home environment, there is a need for further research to establish optimal eligibility criteria to promote infant safety.

Further, primary care providers supporting families receiving home weaning need protocols in place to guide decision making regarding referral to other disciplines (eg, physicians, social work, lactation specialists, nurses, etc.). Given that infants and families undergoing home weaning for NAS require both pharmacologic treatment and social follow up, there is a role for primary healthcare providers to assume the care of infants receiving outpatient weaning for NAS. However, this role requires further development to ensure infant safety and well-being.

### Implications for future research

Findings from this systematic review have several research implications. Larger, prospective studies are required to rigorously assess the effectiveness and safety of home weaning. Further research is also required to identify whether there is an optimal protocol to guide treatment with an emphasis on evaluating duration of treatment, cumulative dose and safety (eg, adverse events). Moreover, consideration should be given to the challenges of identifying which NAS outcomes are important to measure. Improving length of stay is important from a cost perspective; however, future research is needed to conduct robust economic evaluations to ascertain healthcare savings for infants weaned at home. Furthermore, duration of treatment and cumulative dose have potential negative impacts on infants and must be considered. Further exploration of eligibility criteria for home weaning that optimizes infant safety is also necessary before recommending this treatment approach. In addition, given that home weaning is a relatively new practice, qualitative research that explores the experiences of mothers is needed to understand mother’s perception of their role in outpatient weaning, effectiveness of supports, and efficacy in providing this treatment in a home environment. Finally, further research is required on the effect of the involvement of a multi-disciplinary team on outpatient weaning.

## Conclusion

The findings from this systematic review suggest that outpatient weaning for select infants with NAS was effective in reducing the length of hospital stay with minimal adverse outcomes or need for child welfare involvement. However, the duration of pharmacologic treatment was typically longer for infants weaned at home and warrants further evaluation due to the unknown long-term effects of opioids on infants. Furthermore, given the reduction in the length of hospital stay, outpatient weaning may be effective in reducing related healthcare costs, however, large-scale trials are required to establish cost-effectiveness.
